# A new diagnostic method using air perfusion radiography under DSA for gastric stump‐pleural fistula: Report of five cases

**DOI:** 10.1111/1759-7714.13132

**Published:** 2019-07-03

**Authors:** Tao Wu, Ruimin Sun, Mingqin Zhang, Niu Miao, Huili Wang, Dongyi Yang, Yangyang Liu, Qing Zhou

**Affiliations:** ^1^ Department of Radiology Intervention The First Affiliated Hospital of Henan University of Traditional Chinese Medicine (TMC) Zhengzhou China; ^2^ Department of Radiology Intervention The First Affiliated Hospital of Henan University Kaifeng China

**Keywords:** Diagnosis, DSA, gastric stump‐pleural fistula, radiography

## Abstract

Gastric stump‐pleural fistula is a common complication following gastroesophageal anastomosis in patients diagnosed with gastric cancer. Mortality is high because of the severe subsequent relevant complications caused by the fistula. Here we report five cases of gastric stump‐pleural fistula diagnosed by air perfusion radiography under digital subtraction angiography (DSA). DSA air perfusion radiography provides a reliable basis for the development of clinical programmes; it is a simple method which does not involve any pain or trauma to the patient.

## Introduction

Gastroesophageal anastomosis is a conventional surgical method of treatment which may occasionally cause gastric stump fistula followed by systemic symptoms.^1^ Gastric stump‐pleural fistula has a high mortality rate because there may be subsequent complications caused by the fistula.^2^ Early detection of gastric stump fistula will help to prevent fistula‐related systemic symptoms.^3^ Routine X‐ray barium meal, oral methylene blue and CT examination are helpful in establishing a diagnosis. However, it may be difficult to detect the location and size of the fistula and easy to misdiagnosis it as an anastomotic fistula.^3^, ^4^ In addition, endoscopy can also be used to determine the presence of a fistula, but endoscopy whilst identifying a fistula may cause great pain with the risk of rupture if the patient has recently undergone gastroesophageal anastomosis.^5^, ^6^ Air perfusion radiography under digital subtraction angiography (DSA) was carried out in patients with a suspected fistula in order to quickly and easily reach a diagnosis and reduce pain.

## Methods

A total of five patients (two males and three females) were studied in the Department of Interventional Radiology of our hospital from January 2013 to December 2017. Of these five cases, three patients were diagnosed with cardiac cancer, and two with esophageal cancer. The age of those included was between 52 to 66 years (average age 60 years). All patients presented with high fever, dyspnea, rapid pulse and empyema. Food debris was also found following chest drainage.

Before DSA air perfusion contrast was undertaken, patients were fully briefed with regard to the purpose and procedure of the DSA air perfusion contrast and an iodine allergy test was carried out. For those patients with a nasogastric tube, air was injected through this into the remnant stomach. For patients without a nasogastric tube, air was injected into a catheter inserted into the remnant stomach before DSA air perfusion contrast. Under fluoroscopic observation of the position of the epiglottis and dynamic monitoring, a 0.035 in guide wire with a 4F VER catheter was inserted through the oral or nasal cavity into the esophagus by asking the patient to swallow and adjusting the orientation of the catheter tip towards the patient's foot on that side. After the guide wire was inserted into the chest segment of the esophagus, the guide wire was exited and 1 mL of angiography contrast agent injected into the catheter to confirm the esophageal position of catheter. Finally, the guide wire was reinserted into the catheter to guide the catheter into the remnant stomach.

**Figure 1:**
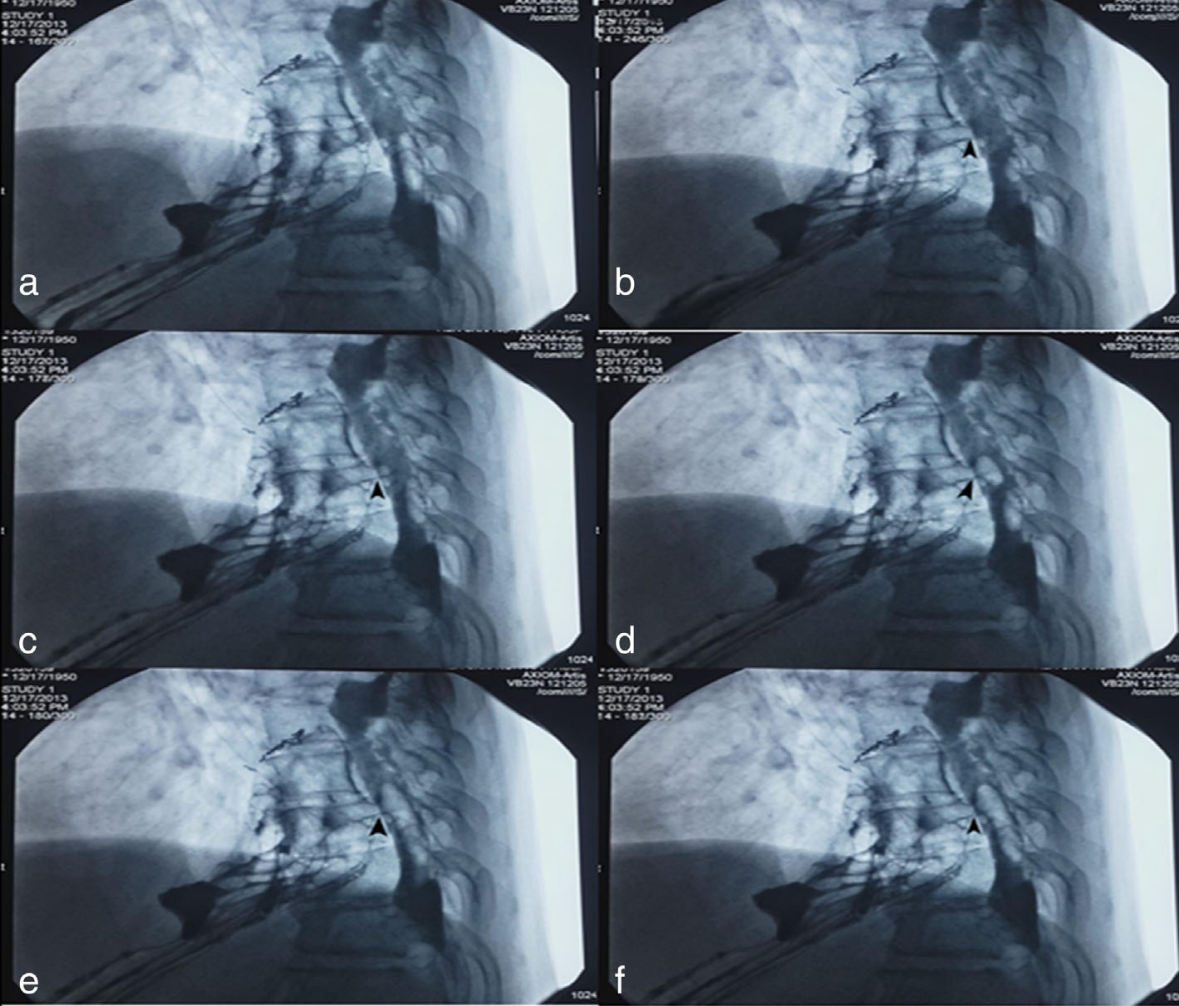
DSA air perfusion contrast was performed which diagnosed stump‐pleural fistula in a 63‐year‐old patient with cardiac cancer. (a).Esophageography showed the fistula formation, but could not the fistula formation, but could not really identify its location. (b) Black arrowhead indicates that air flowed into the fistula tract. (c) A little round bubble was found which confirmed the air flowed into the pleura through the fistula tract. (d, e) The size of the bubbles increased and they changed in shape. (f) An isolated bubble image was found.

Following the operative procedure of DSA air perfusion contrast and oral administration of 10 mL iodine as a contrast agent, the patient was asked to lay flat on their back on an operating room bed with DSA detectors. The flow of contrast agent to the stomach was monitored, and the location of the fistula or suspected fistula point detected by DSA under multi‐angle observations, and the default observation position as a target location of fistula determined. Under the DSA monitoring of the target location, 60 mL of air was injected into the nasogastric tube (or preplaced catheter) and the patient was asked to breathe deeply, exhale from the lungs or cough to increase abdominal pressure. Air leak and the location of air bubbles into the chest were detected by the dynamic multi‐angle observation of DSA. The target location of fistula was reconfirmed under the observation of DSA, the size of the fistula determined by tangent‐bit images and the orientation of the fistula determined by rotation angles of DSA (Fig 1). The whole operation procedure was videoed and the study approved by the Ethics Committee of the hospital. All cases were diagnosed quickly using this approach, and all images clearly showed the size and position of the fistula.

## Discussion

Our results show that gastric stump‐pleural fistula can be diagnosed by DSA air perfusion contrast in a timely, clear and intuitive manner. DSA air perfusion contrast examination was based the leakage and going up of air by using the conventional x‐ray examination method, and DSA has the advantage of multi‐angle observations.

A total of 10 mL contrast agent of iodine was injected into the stomach cavity to view the direction of the bubble flow and form a visual contrast. However, the high dose of iodinated contrast concealed the observation and measurement of the fistula revealed by bubbles. After the rapid injection of air, the residual stomach cavity becomes a relatively closed container with high pressure of air. In the case of deep breathing, expiration or coughing which increases abdominal pressure, the remnant stomach will contract followed by an increase of its inner pressure. The air in the remnant stomach with high inner pressure will be gradually discharged through the fistula with bubble formation. The process of the formation of bubbles by the air extrusion deformation and discharge from the fistula and bubbles entering into the chest can be monitored by DSA multi‐angle observations. The dynamic process of air entering from the remnant stomach into chest confirms the presence of thoracic gastric fistula. By combining with the rotation angles of DSA, the fistula location, its relationship with the surrounding tissue and the size of fistula can be determined. This provides clinicians with a reliable basis for the rational development of treatment programmes by presenting the fistula information visually. However, the amount of air perfusion should not be too much to affect the visual contrast of iodine.

## Conclusion

DSA air perfusion contrast provides a reliable basis for the development of a clinical programme and this method is simple without pain and trauma. However, the clinical application value of this diagnostic procedure requires further confirmation as only five patients were included in this report.
